# (*Z*)-4-[(2-Amino-4,5-dichloro­anilino)(phenyl)methyl­idene]-3-methyl-1-phenyl-1*H*-pyrazol-5(4*H*)-one

**DOI:** 10.1107/S160053681204086X

**Published:** 2012-10-20

**Authors:** Dan Zou, Xingqiang Lü, Shunsheng Zhao, Xiangrong Liu

**Affiliations:** a College of Chemical Engineering, Northwest University, Xi’an 710069, Shannxi, People’s Republic of China; b College of Chemistry and Chemical Engineering, Xian University of Science and Technology, Xi’an 710054, Shannxi, People’s Republic of China

## Abstract

The mol­ecule of the title compound, C_23_H_18_Cl_2_N_4_O, assumes a non-planar conformation in which the pyrazolone ring forms dihedral angles of 32.61 (19), 76.73 (14) and 52.57 (19)° with the three benzene rings. The secondary amino group is involved in an intra­molecular N—H⋯O hydrogen bond. In the crystal, mol­ecules are linked by pairs of N—H⋯O hydrogen bonds, forming inversion dimers. An offset stacking inter­action is observed between the chloro-substituted benzene rings protruding on both sides of these dimers [centroid–centroid distance = 3.862 (1) Å].

## Related literature

For related structures, see: Lu *et al.* (2011 ▶). For bond-length data, see: Allen *et al.* (1987 ▶). For the catalytic properties of asymmetric Schiff bases, see: Caboni *et al.* (2012 ▶). For the synthesis, see: Hennig & Mann (1988 ▶).
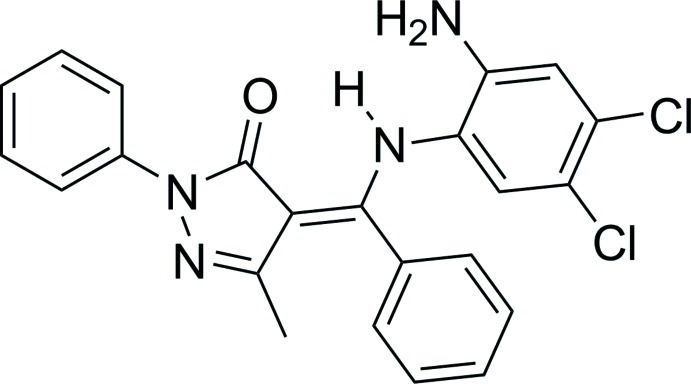


## Experimental

### 

#### Crystal data

C_23_H_18_Cl_2_N_4_O
*M* *_r_* = 437.31
Triclinic, 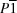

*a* = 8.0653 (16) Å
*b* = 10.931 (2) Å
*c* = 13.851 (3) Å
α = 111.627 (3)°
β = 90.775 (3)°
γ = 110.226 (3)°
*V* = 1051.1 (4) Å^3^
*Z* = 2
Mo *K*α radiation
μ = 0.33 mm^−1^
*T* = 296 K
0.30 × 0.21 × 0.18 mm

#### Data collection

Bruker SMART 1K CCD area-detector diffractometer
Absorption correction: multi-scan (*SADABS*; Sheldrick, 2004 ▶) *T*_min_ = 0.857, *T*_max_ = 1.000
5308 measured reflections
3688 independent reflections
1992 reflections with *I* > 2σ(*I*)
*R*_int_ = 0.027

#### Refinement

*R*[*F* ^2^ > 2σ(*F* ^2^)] = 0.060
*wR*(*F* ^2^) = 0.189
*S* = 1.03
3688 reflections
280 parameters
2 restraints
H atoms treated by a mixture of independent and constrained refinement
Δρ_max_ = 0.51 e Å^−3^
Δρ_min_ = −0.45 e Å^−3^

### 

Data collection: *SMART* (Bruker, 2001 ▶); cell refinement: *SAINT* (Bruker, 2001 ▶); data reduction: *SAINT*; program(s) used to solve structure: *SHELXS97* (Sheldrick, 2008 ▶); program(s) used to refine structure: *SHELXL97* (Sheldrick, 2008 ▶); molecular graphics: *SHELXTL* (Sheldrick, 2008 ▶); software used to prepare material for publication: *SHELXTL* and local programs.

## Supplementary Material

Crystal structure: contains datablock(s) I, global. DOI: 10.1107/S160053681204086X/fy2067sup1.cif

Structure factors: contains datablock(s) I. DOI: 10.1107/S160053681204086X/fy2067Isup2.hkl

Supplementary material file. DOI: 10.1107/S160053681204086X/fy2067Isup3.cml

Additional supplementary materials:  crystallographic information; 3D view; checkCIF report

## Figures and Tables

**Table 1:** Hydrogen-bond geometry (Å, °)

*D*—H⋯*A*	*D*—H	H⋯*A*	*D*⋯*A*	*D*—H⋯*A*
N3—H3*A*⋯O1	0.86	2.01	2.735 (4)	141
N4—H4*B*⋯O1^i^	0.88 (2)	2.18 (2)	3.022 (5)	162 (4)

Symmetry code: (i) 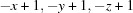.

**Fig. 1.**
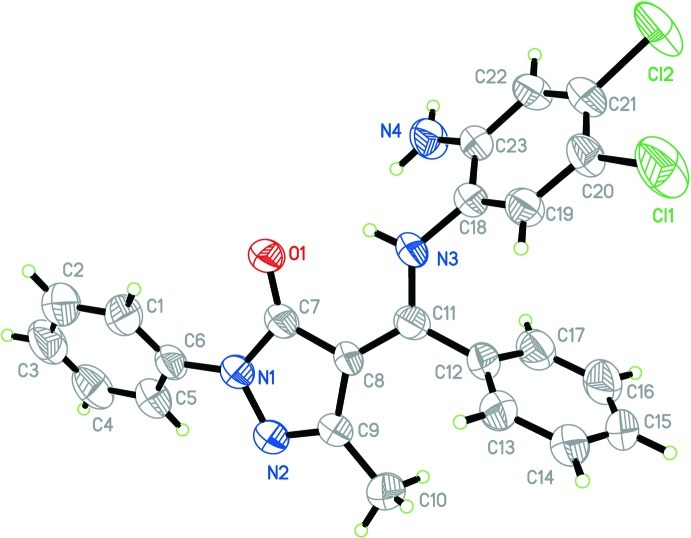
The molecular structure of (I), with atom labels and 50% probability displacement ellipsoids for non-H atoms.

**Fig. 2.**
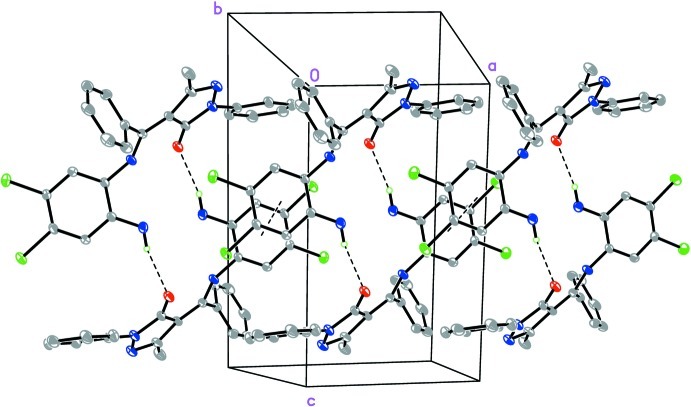
The packing of (I), showing molecules connected by hydrogen bonds (dashed lines) and stacking interaction. H atoms not involved in hydrogen bonding have been omitted.

## supplementary crystallographic information

### Comment

Asymmetrical Schiff bases are of interest due to their catalytic activity and the selectivity of their transition metal complexes in various reactions. Several asymmetrical Schiff base ligands and their transition metal complexes have been synthesized and studied. Here we report the crystal structure of a novel asymmetrical Schiff base ligand (Fig. 1). Bond lengths of the compound are in the range of normal values (Allen *et al.*, 1987) and are comparable to those observed in similar compounds (Lu *et al.*, 2011). The molecules are linked by N—H···O hydrogen bonds and stacking interaction, as shown in Fig. 2. The distance between the centroids of adjacent rings (C18 to C23, *x*, *y*, *z* and -*x* + 2, -*y* + 1, -*z* + 1) is 3.862 (1) Å.

### Experimental

The title compound was obtained according to the synthetic procedure of Hennig & Mann (1988) with some modification. 1,2-diamino-4,5-dichlorobenzene and 4-benzoyl-3-methyl-1-phenyl-1*H*-pyrazol-5(4*H*)-one were refluxed for 2 h in a molar ratio of 1:1 in absolute ethanol to give the product. The single-crystal suitble for X-ray diffraction was obtained by slow evaporation of the ethanolic solution of the title compound.

### Refinement

H atoms of –NH_2_ group were located from a difference map and refined with a distance restraint of N—H = 0.87 (2) Å. Other H atoms were positioned geometrically and refined using a riding model with C—H = 0.95–0.99 Å, and with *U*_iso_(H) = 1.2 (1.5 for methyl groups) times *U*_eq_(C/N). The reflection -2 1 1 is a strong outlier and was omitted in the refinement.

### Crystal data

**Table:**

C_23_H_18_Cl_2_N_4_O	*Z* = 2
*M**_r_* = 437.31	*F*(000) = 452
Triclinic, *P*1	*D*_x_ = 1.382 Mg m^−^^3^
Hall symbol: -P 1	Mo *K*α radiation, λ = 0.71073 Å
*a* = 8.0653 (16) Å	Cell parameters from 3250 reflections
*b* = 10.931 (2) Å	θ = 1.8–25.2°
*c* = 13.851 (3) Å	µ = 0.33 mm^−^^1^
α = 111.627 (3)°	*T* = 296 K
β = 90.775 (3)°	Block, red
γ = 110.226 (3)°	0.30 × 0.21 × 0.18 mm
*V* = 1051.1 (4) Å^3^	

### Data collection

**Table:**

Bruker SMART 1K CCD area-detector diffractometer	3688 independent reflections
Radiation source: fine-focus sealed tube	1992 reflections with *I* > 2σ(*I*)
Graphite monochromator	*R*_int_ = 0.027
thin–slice ω scans	θ_max_ = 25.1°, θ_min_ = 2.1°
Absorption correction: multi-scan (*SADABS*; Sheldrick, 2004)	*h* = −9→7
*T*_min_ = 0.857, *T*_max_ = 1.000	*k* = −13→12
5308 measured reflections	*l* = −15→16

### Refinement

**Table:**

Refinement on *F*^2^	Primary atom site location: structure-invariant direct methods
Least-squares matrix: full	Secondary atom site location: difference Fourier map
*R*[*F*^2^ > 2σ(*F*^2^)] = 0.060	Hydrogen site location: inferred from neighbouring sites
*wR*(*F*^2^) = 0.189	H atoms treated by a mixture of independent and constrained refinement
*S* = 1.03	*w* = 1/[σ^2^(*F*_o_^2^) + (0.0881*P*)^2^] where *P* = (*F*_o_^2^ + 2*F*_c_^2^)/3
3688 reflections	(Δ/σ)_max_ < 0.001
280 parameters	Δρ_max_ = 0.51 e Å^−^^3^
2 restraints	Δρ_min_ = −0.45 e Å^−^^3^

### Special details

**Table:**

Geometry. All e.s.d.'s (except the e.s.d. in the dihedral angle between two l.s. planes) are estimated using the full covariance matrix. The cell e.s.d.'s are taken into account individually in the estimation of e.s.d.'s in distances, angles and torsion angles; correlations between e.s.d.'s in cell parameters are only used when they are defined by crystal symmetry. An approximate (isotropic) treatment of cell e.s.d.'s is used for estimating e.s.d.'s involving l.s. planes.
Refinement. Refinement of *F*^2^ against ALL reflections. The weighted *R*-factor *wR* and goodness of fit *S* are based on *F*^2^, conventional *R*-factors *R* are based on *F*, with *F* set to zero for negative *F*^2^. The threshold expression of *F*^2^ > σ(*F*^2^) is used only for calculating *R*-factors(gt) *etc*. and is not relevant to the choice of reflections for refinement. *R*-factors based on *F*^2^ are statistically about twice as large as those based on *F*, and *R*- factors based on ALL data will be even larger.

### Fractional atomic coordinates and isotropic or equivalent isotropic displacement parameters (Å^2^)

**Table:**

	*x*	*y*	*z*	*U*_iso_*/*U*_eq_	
Cl2	1.11130 (17)	0.22914 (15)	0.36132 (9)	0.0878 (5)	
Cl1	1.18254 (17)	0.24718 (17)	0.59173 (10)	0.0939 (5)	
O1	0.5427 (3)	0.6431 (3)	0.7490 (2)	0.0541 (7)	
N2	0.2712 (4)	0.4857 (3)	0.8999 (2)	0.0573 (9)	
N3	0.6743 (4)	0.4320 (3)	0.6750 (2)	0.0498 (8)	
H3A	0.6612	0.5073	0.6759	0.060*	
N1	0.3415 (4)	0.5927 (3)	0.8613 (2)	0.0511 (8)	
C19	0.9177 (5)	0.3465 (4)	0.6293 (3)	0.0503 (10)	
H19A	0.9434	0.3579	0.6985	0.060*	
C22	0.8476 (5)	0.3187 (4)	0.4243 (3)	0.0502 (10)	
H22A	0.8266	0.3111	0.3558	0.060*	
C18	0.7801 (5)	0.3803 (4)	0.6009 (3)	0.0433 (9)	
C9	0.3620 (5)	0.4033 (4)	0.8684 (3)	0.0532 (10)	
C6	0.2573 (5)	0.6900 (4)	0.8746 (3)	0.0482 (9)	
C23	0.7409 (5)	0.3646 (4)	0.4970 (3)	0.0451 (9)	
C11	0.5925 (5)	0.3812 (4)	0.7431 (3)	0.0444 (9)	
C7	0.4691 (5)	0.5709 (4)	0.8000 (3)	0.0455 (9)	
C20	1.0185 (5)	0.2954 (4)	0.5552 (3)	0.0526 (10)	
C12	0.6100 (5)	0.2515 (4)	0.7451 (3)	0.0459 (9)	
N4	0.6018 (5)	0.3963 (4)	0.4673 (3)	0.0624 (10)	
C8	0.4918 (5)	0.4480 (4)	0.8058 (3)	0.0444 (9)	
C21	0.9834 (5)	0.2847 (4)	0.4539 (3)	0.0538 (10)	
C13	0.7013 (5)	0.2581 (4)	0.8339 (3)	0.0537 (10)	
H13A	0.7464	0.3428	0.8934	0.064*	
C5	0.0782 (6)	0.6554 (5)	0.8833 (3)	0.0607 (11)	
H5A	0.0107	0.5666	0.8825	0.073*	
C1	0.3573 (6)	0.8244 (4)	0.8808 (3)	0.0623 (11)	
H1A	0.4793	0.8504	0.8782	0.075*	
C17	0.5389 (5)	0.1236 (4)	0.6589 (3)	0.0625 (11)	
H17A	0.4743	0.1174	0.6000	0.075*	
C15	0.6556 (7)	0.0121 (5)	0.7463 (4)	0.0749 (14)	
H15A	0.6714	−0.0683	0.7462	0.090*	
C10	0.3125 (6)	0.2748 (4)	0.8947 (4)	0.0731 (14)	
H10A	0.2210	0.2745	0.9383	0.110*	
H10B	0.4161	0.2775	0.9316	0.110*	
H10C	0.2687	0.1904	0.8310	0.110*	
C4	−0.0012 (6)	0.7519 (6)	0.8933 (3)	0.0719 (13)	
H4C	−0.1228	0.7268	0.8969	0.086*	
C2	0.2773 (7)	0.9199 (5)	0.8910 (4)	0.0775 (14)	
H2B	0.3445	1.0095	0.8932	0.093*	
C14	0.7249 (6)	0.1390 (5)	0.8336 (4)	0.0668 (12)	
H14A	0.7879	0.1441	0.8926	0.080*	
C16	0.5628 (6)	0.0062 (5)	0.6596 (4)	0.0753 (13)	
H16A	0.5157	−0.0789	0.6006	0.090*	
C3	0.0976 (8)	0.8833 (6)	0.8979 (4)	0.0811 (15)	
H3B	0.0442	0.9486	0.9057	0.097*	
H4A	0.508 (4)	0.381 (4)	0.495 (3)	0.062 (13)*	
H4B	0.572 (6)	0.371 (5)	0.3997 (17)	0.092 (17)*	

### Atomic displacement parameters (Å^2^)

**Table:**

	*U*^11^	*U*^22^	*U*^33^	*U*^12^	*U*^13^	*U*^23^
Cl2	0.0813 (9)	0.1355 (12)	0.0620 (7)	0.0711 (9)	0.0337 (6)	0.0267 (7)
Cl1	0.0773 (9)	0.1571 (13)	0.0793 (9)	0.0818 (9)	0.0190 (7)	0.0461 (9)
O1	0.0631 (17)	0.0617 (17)	0.0604 (17)	0.0372 (14)	0.0316 (13)	0.0354 (14)
N2	0.070 (2)	0.059 (2)	0.060 (2)	0.0348 (19)	0.0336 (17)	0.0300 (17)
N3	0.062 (2)	0.058 (2)	0.0479 (19)	0.0391 (17)	0.0273 (16)	0.0249 (16)
N1	0.057 (2)	0.0536 (19)	0.058 (2)	0.0311 (17)	0.0300 (16)	0.0295 (16)
C19	0.053 (2)	0.059 (2)	0.043 (2)	0.026 (2)	0.0123 (18)	0.0201 (19)
C22	0.048 (2)	0.065 (3)	0.041 (2)	0.026 (2)	0.0137 (18)	0.0214 (19)
C18	0.044 (2)	0.048 (2)	0.046 (2)	0.0230 (18)	0.0170 (17)	0.0215 (17)
C9	0.067 (3)	0.056 (2)	0.051 (2)	0.032 (2)	0.028 (2)	0.027 (2)
C6	0.056 (3)	0.056 (2)	0.045 (2)	0.035 (2)	0.0187 (18)	0.0200 (19)
C23	0.049 (2)	0.048 (2)	0.043 (2)	0.0229 (19)	0.0135 (17)	0.0178 (18)
C11	0.046 (2)	0.051 (2)	0.040 (2)	0.0218 (19)	0.0120 (17)	0.0200 (18)
C7	0.046 (2)	0.051 (2)	0.045 (2)	0.0242 (19)	0.0168 (18)	0.0194 (18)
C20	0.046 (2)	0.066 (3)	0.051 (2)	0.031 (2)	0.0133 (19)	0.020 (2)
C12	0.047 (2)	0.046 (2)	0.049 (2)	0.0238 (19)	0.0154 (18)	0.0182 (19)
N4	0.060 (3)	0.088 (3)	0.067 (3)	0.047 (2)	0.021 (2)	0.042 (2)
C8	0.057 (2)	0.047 (2)	0.042 (2)	0.0327 (19)	0.0211 (18)	0.0192 (17)
C21	0.045 (2)	0.064 (3)	0.053 (2)	0.025 (2)	0.0189 (19)	0.019 (2)
C13	0.067 (3)	0.052 (2)	0.047 (2)	0.028 (2)	0.012 (2)	0.0184 (19)
C5	0.060 (3)	0.065 (3)	0.057 (3)	0.030 (2)	0.018 (2)	0.018 (2)
C1	0.061 (3)	0.065 (3)	0.073 (3)	0.034 (2)	0.024 (2)	0.031 (2)
C17	0.069 (3)	0.058 (3)	0.055 (3)	0.025 (2)	0.018 (2)	0.016 (2)
C15	0.109 (4)	0.071 (3)	0.087 (4)	0.064 (3)	0.056 (3)	0.046 (3)
C10	0.093 (4)	0.066 (3)	0.088 (3)	0.043 (3)	0.051 (3)	0.048 (3)
C4	0.059 (3)	0.098 (4)	0.064 (3)	0.047 (3)	0.019 (2)	0.021 (3)
C2	0.098 (4)	0.076 (3)	0.083 (3)	0.054 (3)	0.037 (3)	0.037 (3)
C14	0.085 (3)	0.078 (3)	0.067 (3)	0.053 (3)	0.032 (2)	0.039 (3)
C16	0.096 (4)	0.048 (3)	0.075 (3)	0.029 (3)	0.032 (3)	0.015 (2)
C3	0.110 (4)	0.103 (4)	0.066 (3)	0.080 (4)	0.033 (3)	0.035 (3)

### Geometric parameters (Å, º)

**Table:**

Cl2—C21	1.724 (4)	C12—C17	1.380 (5)
Cl1—C20	1.716 (4)	C12—C13	1.391 (5)
O1—C7	1.254 (4)	N4—H4A	0.850 (18)
N2—C9	1.309 (4)	N4—H4B	0.876 (19)
N2—N1	1.409 (4)	C13—C14	1.378 (5)
N3—C11	1.336 (4)	C13—H13A	0.9300
N3—C18	1.424 (4)	C5—C4	1.380 (5)
N3—H3A	0.8600	C5—H5A	0.9300
N1—C7	1.372 (4)	C1—C2	1.374 (5)
N1—C6	1.409 (4)	C1—H1A	0.9300
C19—C18	1.379 (5)	C17—C16	1.365 (5)
C19—C20	1.394 (5)	C17—H17A	0.9300
C19—H19A	0.9300	C15—C16	1.375 (6)
C22—C21	1.374 (5)	C15—C14	1.380 (6)
C22—C23	1.398 (5)	C15—H15A	0.9300
C22—H22A	0.9300	C10—H10A	0.9600
C18—C23	1.404 (5)	C10—H10B	0.9600
C9—C8	1.439 (5)	C10—H10C	0.9600
C9—C10	1.502 (5)	C4—C3	1.357 (7)
C6—C1	1.379 (5)	C4—H4C	0.9300
C6—C5	1.380 (5)	C2—C3	1.378 (7)
C23—N4	1.381 (5)	C2—H2B	0.9300
C11—C8	1.382 (5)	C14—H14A	0.9300
C11—C12	1.482 (5)	C16—H16A	0.9300
C7—C8	1.448 (5)	C3—H3B	0.9300
C20—C21	1.380 (5)		
			
C9—N2—N1	105.9 (3)	C11—C8—C7	122.6 (3)
C11—N3—C18	129.7 (3)	C9—C8—C7	104.5 (3)
C11—N3—H3A	115.2	C22—C21—C20	121.3 (3)
C18—N3—H3A	115.2	C22—C21—Cl2	118.2 (3)
C7—N1—C6	128.5 (3)	C20—C21—Cl2	120.5 (3)
C7—N1—N2	112.0 (3)	C14—C13—C12	119.9 (4)
C6—N1—N2	118.6 (3)	C14—C13—H13A	120.1
C18—C19—C20	120.5 (3)	C12—C13—H13A	120.1
C18—C19—H19A	119.7	C6—C5—C4	120.3 (4)
C20—C19—H19A	119.7	C6—C5—H5A	119.8
C21—C22—C23	120.2 (3)	C4—C5—H5A	119.8
C21—C22—H22A	119.9	C2—C1—C6	120.1 (4)
C23—C22—H22A	119.9	C2—C1—H1A	119.9
C19—C18—C23	120.3 (3)	C6—C1—H1A	119.9
C19—C18—N3	121.7 (3)	C16—C17—C12	120.4 (4)
C23—C18—N3	118.0 (3)	C16—C17—H17A	119.8
N2—C9—C8	112.2 (3)	C12—C17—H17A	119.8
N2—C9—C10	118.4 (3)	C16—C15—C14	119.1 (4)
C8—C9—C10	129.3 (3)	C16—C15—H15A	120.4
C1—C6—C5	119.1 (4)	C14—C15—H15A	120.4
C1—C6—N1	119.1 (4)	C9—C10—H10A	109.5
C5—C6—N1	121.8 (4)	C9—C10—H10B	109.5
N4—C23—C22	120.6 (3)	H10A—C10—H10B	109.5
N4—C23—C18	120.7 (3)	C9—C10—H10C	109.5
C22—C23—C18	118.6 (3)	H10A—C10—H10C	109.5
N3—C11—C8	119.3 (3)	H10B—C10—H10C	109.5
N3—C11—C12	117.7 (3)	C3—C4—C5	120.2 (4)
C8—C11—C12	123.0 (3)	C3—C4—H4C	119.9
O1—C7—N1	125.3 (3)	C5—C4—H4C	119.9
O1—C7—C8	129.5 (3)	C1—C2—C3	120.2 (5)
N1—C7—C8	105.2 (3)	C1—C2—H2B	119.9
C21—C20—C19	119.0 (3)	C3—C2—H2B	119.9
C21—C20—Cl1	121.7 (3)	C13—C14—C15	120.5 (4)
C19—C20—Cl1	119.3 (3)	C13—C14—H14A	119.8
C17—C12—C13	119.1 (3)	C15—C14—H14A	119.8
C17—C12—C11	120.8 (3)	C17—C16—C15	120.9 (4)
C13—C12—C11	120.0 (3)	C17—C16—H16A	119.5
C23—N4—H4A	120 (3)	C15—C16—H16A	119.5
C23—N4—H4B	117 (3)	C4—C3—C2	120.0 (4)
H4A—N4—H4B	110 (4)	C4—C3—H3B	120.0
C11—C8—C9	131.6 (3)	C2—C3—H3B	120.0

### Hydrogen-bond geometry (Å, º)

**Table:**

*D*—H···*A*	*D*—H	H···*A*	*D*···*A*	*D*—H···*A*
N3—H3*A*···O1	0.86	2.01	2.735 (4)	141
N4—H4*B*···O1^i^	0.88 (2)	2.18 (2)	3.022 (5)	162 (4)

Symmetry code: (i) −*x*+1, −*y*+1, −*z*+1.
